# CIHR canadian HIV trials network HIV workshop: ethical research through community participation and strengthening scientific validity

**DOI:** 10.11604/pamj.2014.19.44.4766

**Published:** 2014-09-19

**Authors:** Lawrence Mbuagbaw, Amy Slogrove, Jacqueline Sas, John Kunda, Frederick Morfaw, Jackson Mukonzo, Lehana Thabane

**Affiliations:** 1 Department of Clinical Epidemiology and Biostatistics, McMaster University, Hamilton, ON, Canada; 2 Biostatistics Unit, Father Sean O'Sullivan Research Centre, St Joseph's Healthcare—Hamilton, ON, Canada; 3 Centre for Development of Best Practices in Health, Yaoundé Central Hospital, Yaoundé, Cameroon; 4 Department of Paediatrics & Child Health, Faculty of Medicine and Health Sciences, Stellenbosch University, South Africa; 5 School of Population and Public Health, University of British Columbia, Vancouver, Canada; 6 CIHR Canadian HIV Trials Network, University of British Columbia, Canada; 7 Community Information and Epidemiological Technologies, Lusaka, Zambia; 8 Department of Obstetrics and Gynaecology, Faculty of Medicines and Biomedical Sciences, University of Yaoundé 1, Yaoundé, Cameroon; 9 School of Biomedical Sciences, College of Health Sciences, University of Makerere, Kampala, Uganda; 10 Departments of Paediatrics and Anaesthesia, McMaster University, Hamilton, ON, Canada; 11 Centre for Evaluation of Medicine, St Joseph's Healthcare-Hamilton, ON, Canada; 12 Population Health Research Institute, Hamilton Health Sciences, Hamilton, ON, Canada

**Keywords:** Capacity building, ethics, HIV research, South Africa

## Abstract

The CIHR canadian HIV trials network mandate includes strengthening capacity to conduct and apply clinical research through training and mentoring initiatives of HIV researchers by building strong networks and partnerships on the African continent. At the17th International Conference on AIDS and Sexually Transmitted Infections in Africa (ICASA), the CTN facilitated a two-day workshop to address ethical issues in the conduct of HIV research, and career enhancing strategies for young African HIV researchers. Conference attendees were allowed to attend whichever session was of interest to them. We report on the topics covered, readings shared and participants’ evaluation of the workshop. The scientific aspects of ethical research in HIV and career enhancement strategies are relevant issues to conference attendees.

## Introduction

The CTN is a Canada-wide partnership of researchers, caregivers, governments, health advocates, the innovative pharmaceutical and biotechnology industry, and people living with HIV who are committed to developing treatments, preventions and a cure for HIV and related health conditions, through the conduct of scientifically sound and ethical trials. Two important elements of CTN's mandate are to train the next generation of HIV clinician researchers which it accomplishes using its successful Postdoctoral Fellowship Awards Program, and establishing partnerships with international researchers and research groups. Their National Centre is located are in Vancouver, British Columbia, Canada. The overarching goal of the CTN International Postdoctoral Fellowship programme is to build mentee skills and capacity to conduct HIV clinical research. More specific goals include career development, building collaborative networks and completing specific research projects.

Based on partnerships developed by the CTN with African researchers and international fellows in Cameroon, Zambia, Uganda and South Africa over the last few years, the CTN has been able to address the growing need to build skills and capacity amongst African researchers with regards to clinical trials methodology, and to maximize their participation in a growing African-Canadian collaborative research community [1]. In line with the proposed theme of the17th International Conference on AIDS and Sexually Transmitted Infections in Africa (ICASA), the CTN aided a two-day workshop to address ethical issues in the conduct of HIV research, and career enhancing strategies for young African HIV researchers.

This workshop addressed limitations in research ethics capacity in Africa that are now prioritized by many international bodies including the World Health Organisation (WHO), Family Health International (FHI), African Malaria Network Trust (AMANET) and many others [2, 3]. We also offered career enhancement strategies based on experience gathered in the CTN international postdoctoral fellowship programme.

## Workshop report

**Location:** the CIHR CTN facilitated a two-day workshop in conjunction with the ICASA conference in Cape Town, South Africa (December 7-11, 2013).

**Aims:** the aims of this workshop were to introduce young HIV researchers to the CIHR Canadian HIV Trials Network, the importance of scientific validity, capacity development and community participation for ethical conduct of HIV research based on the CTN's experience.

**Objectives:** at the end of the course the participants were expected to be able to: 1) Understand the role of the CIHR Canadian HIV Trials Network and its experience in conducting ethically sound HIV research; 2) Understand the importance of scientific validity in ethical research; 3) Understand the importance of capacity building for the conduct of ethically sound HIV research; 4) Acquire knowledge as to ways in which they can enhance their research careers; 5) Describe community involvement in ethical HIV research.

**Participants:** the target audience was primarily young HIV researchers and secondarily, representatives of research institutions, senior researchers (mentors) and members of the community.

**Facilitators:** the facilitators were chosen based on their experience with HIV research in Africa: a professor of biostatistics at McMaster University and Canadian supervisor of four of the five CTN international fellows. The fellows, from Makerere University in Uganda, CIET in Zambia, the University of Yaoundé in Cameroon and the University of Stellenbosch, in South Africa, facilitated some of the sessions, alongside a representative from the CTN.

Pre-workshop tasks: None

**Program:** over two days the participants were introduced to the CTN International Fellowship Programme, the role of mentoring as a means to enhance the ethical conduct of HIV research based on CTN's experience; the place of statistics in the ethical conduct of research; long distance mentoring (day 1); important tips on enhancing a research career; and an ethical perspective on community participation in HIV research (day 2). Each of the talks was independent from the others, so participants were free to choose which talks they would attend.

**Course material and readings:** the participants were provided with reading materials relevant to the topics addressed. Table 1 is a summary of the topics covered in the workshop, the readings and other electronic resources. We sought to make the training relevant to the region by using some local readings and examples as often as possible.

**Table 1:** Workshop outline and resources

Objective	Topics covered	Readings and Internet resources
Understand the role of the CIHR Canadian HIV Trials Network in ethical HIV research	*Introduction to the CIHR CTN and the International postdoctoral fellowship*	CTN Postdoctoral Fellowship Awards Program fellowships: http://www.hivnet.ubc.ca/research-services/postdoctoral-fellowships/
Understand the role of scientific validity in ethical research	*Ethics and scientific validity*	[4–6] Training and resources in research ethics evaluation: http://elearning.trree.org/ Tri-Council Policy Statement 2 online training: http://tcps2core.ca/welcome
Understand the importance of capacity building for ethical conduct of research	*Capacity building for ethical research 1: Long distance mentoring*	[7–9]
List ways in which they can enhance their research careers	*Capacity building for ethical research 2: Tips for enhancing a health research career and building strong collaborations in HIV research*	[9, 10]
Describe community involvement in ethical HIV research	*Community participation in HIV: an ethical perspective (JK)*	[11, 12] CTN Canadian Primer for community Participation: http://www.hivnet.ubc.ca/ctn_hivnet/wp-content/uploads/2010/12/CAC-Primer.pdf

**Evaluation:** participants mostly appreciated the talks on ethics and long-distance mentorship. They also appreciated the consideration of community in research and the practical content of the workshops. Some participants wanted more time for discussion among themselves and more French content. They recommended more handouts, less technical material and more interaction. Table 2: Median ratings (1 = poor; 7= excellent) for evaluation of workshop sessions.

**Table 2:** Median ratings (1 = poor; 7= excellent) for evaluation of workshop sessions

	Session	Item	n	Median( Q1,Q2)
**Day 1**	**Session 1:** *Introduction to the CIHR CTN and the International postdoctoral fellowship*	Clarity of the objectives	38	6.0 (4.0, 6.0)
		Appropriateness of the objectives	34	6.0 (5.0, 6.0)
		Clarity of the presentation	36	6.0 (4.0, 6.0)
		Usefulness of the materials	34	6.0 (4.7, 6.3)
		Overall clarity of presentation	35	6.0 (5.0, 6.0)
	**Session 2:** *Ethics and scientific validity*	Clarity of the objectives	34	6.0 (4.8, 7.0)
		Appropriateness of the objectives	33	6.0 (4.0, 6.0)
		Clarity of the presentation	33	6.0 (4.5, 7.0)
		Usefulness of the materials	32	6.0 (5.0, 6.0)
		Overall clarity of presentation	32	6.0 (5.0, 6.0)
	**Session 3:** *Capacity building for ethical research 1: Long distance mentoring*	Clarity of the objectives	33	6.0 (5.0, 6.0)
		Appropriateness of the objectives	32	6.0 (5.0, 6.0)
		Clarity of the presentation	30	6.0 (5.0, 6.3)
		Usefulness of the materials	30	6.0 (4.0, 6.0)
		Overall clarity of presentation	30	6.0 (5.0, 7.0)
**Day 2**	**Session 4:** *Capacity building for ethical research 2: Tips for enhancing a health research career*	Clarity of the objectives	20	5.0 (4.3, 6.0)
		Appropriateness of the objectives	20	5.0 (5.0, 6.0)
		Clarity of the presentation	20	6.0 (5.0, 6.0)
		Usefulness of the materials	20	5.0 (5.0, 6.0)
		Overall clarity of presentation	18	5.5 (5.0, 6.0)
	**Session 5:** *Community participation in HIV: an ethical perspective*	Clarity of the objectives	17	5.0 (4.0,6.0)
		Appropriateness of the objectives	18	5.0 (5.0, 6.0)
		Clarity of the presentation	18	5.0 (5.0, 6.0)
		Usefulness of the materials	18	5.0 (5.0, 6.0)
		Overall clarity of presentation	18	5.0 (4.0,6.0)

## Conclusion

The workshop received more participants than expected and all sessions were ranked highly. Capacity building in ethical and educational strategies in HIV research was highly appreciated at the ICASA conference. Some additional resources were provided.
